# Discovery of the *Agrobacterium* growth inhibition sequence in virus and its application to recombinant clone screening

**DOI:** 10.1186/s13568-019-0840-3

**Published:** 2019-07-24

**Authors:** Jinlong Yin, Hui Liu, Wenyang Xiang, Tongtong Jin, Dongquan Guo, Liqun Wang, Haijian Zhi

**Affiliations:** 1 National Center for Soybean Improvement, National Key Laboratory for Crop Genetics and Germplasm Enhancement, Nanjing Agricultural University, Weigang 1, Nanjing, 210095 People’s Republic of China; 2 Jilin Academy of Agricultural Sciences, Changchun, 130033 People’s Republic of China

**Keywords:** *Agrobacterium*, Growth inhibition, *Soybean mosaic virus*, Clone screening

## Abstract

Infectious clone vectors used widely in genetic research. While constructing *soybean mosaic virus* (SMV) clone vectors, we found that transformed *Agrobacterium* grew significantly different depending on the viral strains used. In particular, the clone vectors constructed with SMV SC15 significantly suppressed the growth of *Agrobacterium*. Recombinant and truncated virus vector experiments showed that the polymorphism of a P1 protein coding sequence of SC15 leads to the growth inhibition of *Agrobacterium*. But the lack of other protein encoding sequences, except for the sequence encoding coat protein, should reduce the ability of SC15 to suppress *Agrobacterium* growth. A vector (pCB301-*att*L-SC15P) compatible with the Gateway cloning system was constructed using this *Agrobacterium* inhibitory sequence. The results from the LR recombination reaction with pCB301-*att*L-SC15P and *Agrobacterium* transformation showed the valuable application potential of the *Agrobacterium* inhibitory sequence to serve as a negative screening factor for effective recombinant clone screening in *Agrobacterium*.

**Electronic supplementary material:**

The online version of this article (10.1186/s13568-019-0840-3) contains supplementary material, which is available to authorized users.

## Introduction

*Agrobacterium* is the only cellular organism on Earth naturally able to transfer DNA between itself and plants, making it an important tool in plant transgenic techniques, such as genetic transformation and transient expression (Schell and Van 1977; Leuzinger et al. 2013). However, *Agrobacterium* is still rarely used directly as the host cell for recombinant clone screening. Instead, the recombinant clones used in *Agrobacterium* infection experiments usually must be screened in advance in other cells (e.g. *Escherichia coli* and others).

The *soybean mosaic virus* (SMV) belongs to *Potyvirus* genus, whose genome encodes 11 mature proteins: P1, HC-Pro, P3, P3N-PIPO, 6K1, CI, 6K2, NIa-VPg, NIa-Pro, Nib, and CP (Chung et al. 2008; Urcuquiinchima et al. 2001). This virus mainly infects soybean plants and leads to mosaic and necrosis symptoms on leaves (Hill et al. 2007). In China, the SMV isolations have been divided into 21 (SC1 to SC21) strains by 10 different soybean cultivars (Li et al. 2010). Exactly as any other viruses, much of the life history of SMV includes its replication, assembly, and transportation that are achieved by interacting with host effectors. In addition, its transmission can happen by HC-Pro and CP proteins interacting with the putative receptor in aphid stylets (Seo et al. 2010). To date, however, the interaction between any *Potyvirus* and *Agrobacterium* has not been reported on. The P3 and CI proteins of some viruses were reportedly toxic to *E. coli*, but whether there exists a lethal virus protein to *Agrobacterium* remains unknown (Ali et al. 2011; Desbiez et al. 2012).

During our construction of SMV-based clone vectors, we found that one of the SMV physiological strains used, SC15, could significantly inhibit the growth of *Agrobacterium*. Experiments investigating recombination between the SC15 sequences and other strains indicated that the specific sequence underpinning this growth inhibition effect is located in the sequence encoding the P1 protein. While the presence of P1-encoding sequence is not a sufficient condition to inhibit the growth of *Agrobacterium*, the deletion of other sequences will reduce the degree of *Agrobacterium* growth inhibition.

To verify whether or not the *Agrobacterium* inhibitory sequence can be used for screening a recombinant clone in *Agrobacterium*, the vector pCB301-*att*L-SC15P was constructed by inserting this sequence between the *att*L1 and *att*L2 sequences from the Gateway cloning system, and by ligation with the pCB301 frame. The LR recombination reaction was successfully carried out between the donor vectors and pCB301-*att*L-SC15P, and the recombinant reaction product was transformed directly into *Agrobacterium*. The PCR and sequencing results indicated that all the recombinant clones contained in the selected *Agrobacterium* colonies were constructed correct. Perhaps this experimental demonstration will enable us to skip the steps of screening recombination clones with *E. coli* in future *Agrobacterium*-based transformation experiments.

## Materials and methods

### Construction of SMV clone vectors

First, we used TRIZOL method to extract total RNA from fresh soybean leaves infected by SMV. Next, we used PrimeScript II 1st strand cDNA synthesis kit (6210A, TaKaRa, Dalian, China) to carry out reverse translation of total RNA into cDNA. Then, using that cDNA as a template, we used Prime STAR Max DNA Polymerase (R045A, TaKaRa) to amplify five fragments that covered the whole SMV genome, and PCR products were purified with AxyPrep™ DNA Gel Extraction kit (AP-GX-50, Axygen, Wujiang, China). The yeast-*E. coli*–*Agrobacterium* Shuttle Vector pCB301 (Sun et al. 2017) was linearized with *Stu*I and *Sma*I; then, fragments and linearized pCB301 vector were co-transformed into the yeast strain W303-113. After that, the SD/-Trp medium was used to screen the recombinant clones. Finally, the plasmids were extracted from the yeast colony with an E.Z.N.A. Yeast Plasmid Mini Kit (D3376, OMEGA Bio-tek, Norcross, GA, USA) and verified by PCR and sequencing.

The SMV genomic sequences (SC3, SC7 and SC15) used in this experiment were deposited into GenBank with Accession numbers MH919384 to MH919386. All primers used for every vector construction listed in Additional file 1: Table S1.

### Construction of recombinant and truncated clones

Recombinant clone between SC3 and SC15: First, we designed primers with the conserved sequences of the two strains and amplified each of the fragments. Next, we used the Gibson assembly method (Gibson et al. 2009) to assemble proper fragments with a linearized vector and then transformed *E. coli* DH5α. Then, we screened the recombinant clones by PCR and sequencing methods. The Gibson assembly method was also used for the clone construction of truncated fragments.

Recombinant clone between SC7 and SC15: First, we designed primers to obtain the recombinant fragments between P1 and HC-Pro by using overlapping PCR. Then, we used the recombinant fragments together with the rest of the fragments and the linearized vector pCB301 to transform the yeast strain W303-113, the recombinant clones were screened and verified as above.

### Construction of the pCB301-*att*L-SC15P and LR recombination reaction

Constructing pCB301-*att*L-SC15P had two steps. First, we used *att*L1 and *att*L2 and the sequence between them in pGWB5 (Nakagawa et al. 2007) to replace the sequence between LB and RB in pCB301; this product we named pCB301-*att*L-*ccd*B. Next, we used the SC15P fragments to replace the sequence between *att*L1 and *att*L2. PCR amplification was used to obtain the sequences for vector construction, and the Gibson assembly was used for assembling the fragments. The clones were screened in *E. coli* DH5α and verified by PCR and sequencing methods.

The LR recombination reaction (Bushman et al. 1985) was performed separately between the pCB301-*att*L-SC15P plasmids and the pDonor-Zeocin-CI and pDonor-Zeocin-Avh241 plasmids (constructed before in another experiment). Next, 3 μL of the reaction product was directly transformed into *Agrobacterium* EHA105. The clones were verified by PCR and sequencing methods. The primers used for the clones’ PCR were *att*L1-301REC and *att*L2-301REC (Additional file 1: Table S1).

### Transformation, culture, and growth inhibition evaluation of *Agrobacterium*

The *Agrobacterium* strain EHA105 (Hood et al. 1993), GV3101 (Koncz and Schell 1986), LBA4404 (Hoekema et al. 1983) and K599 (Mankin et al. 2007) were used in this experiment. The freeze–thaw method (Weigel and Glazebrook 2006) was used to transform *Agrobacterium*, which was followed by a 2-h recovery period at 28 °C. Then, it was spread on 9-cm-diameter YEP medium (containing 50 ng/mL Kan and 25 ng/mL Rif) and incubated at 28 °C for 48 h. Then their photographs were taken under OLYMPUS MVX10 stereoscopic microscope (Olympus, Tokyo, Japan).

## Results

### *Agrobacterium* growth inhibited by pCB301-SC15

**Fig. 1:**
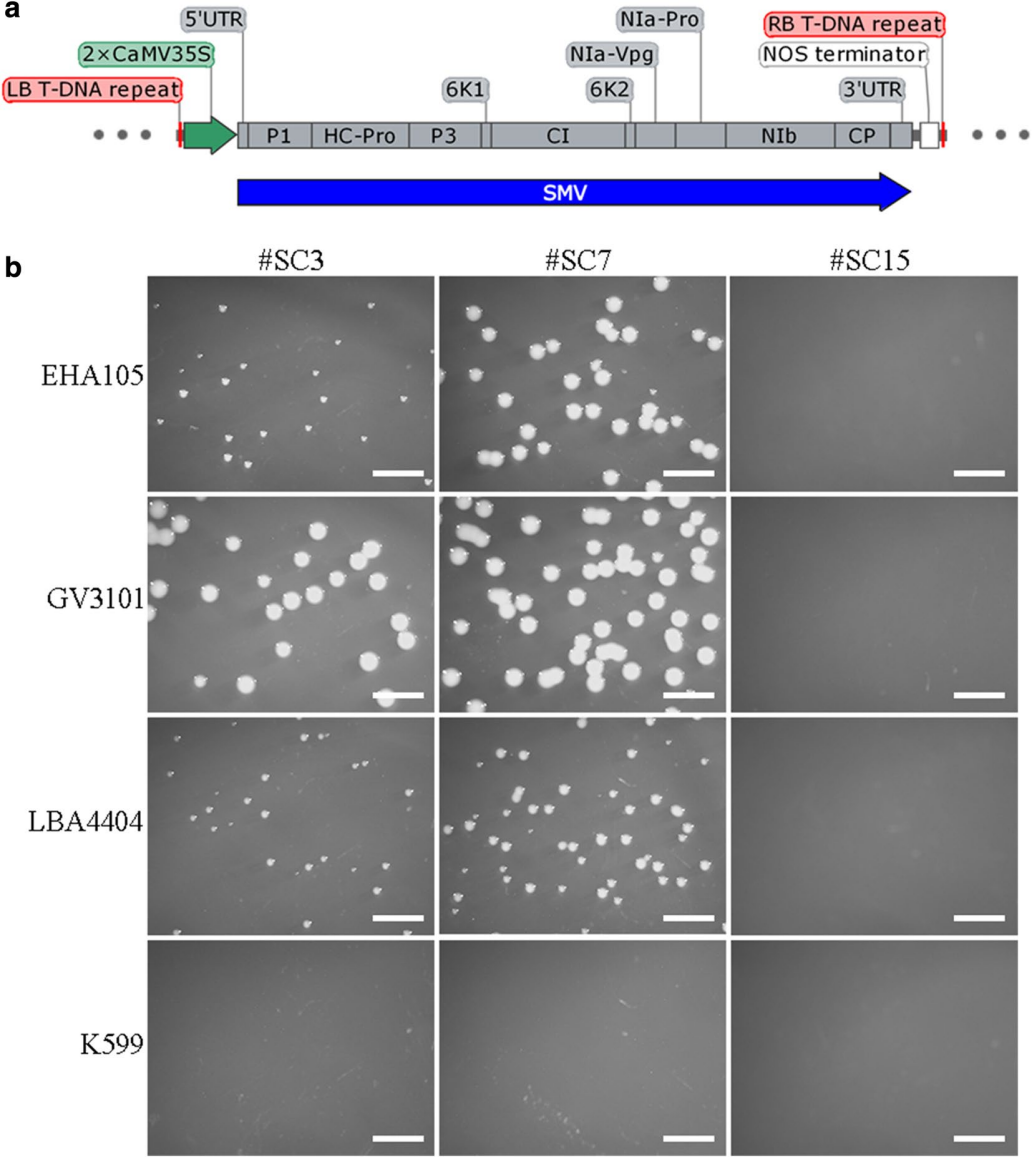
Inhibition of *Agrobacterium* growth by pCB301-SC15. **a** Structure of the virus clone vector, which shows the elements between LB and RB. **b** Growth of *Agrobacterium* after 2 days post-transformation of pCB301-SC3, pCB301-SC7 and pCB301-SC15; scale bars = 4 mm

Virus clone vectors are a prerequisite for genetic studies of RNA viruses. In present study, three SMV clone vectors were constructed (Fig. 1a) and showed excellent infectivity to soybean (*Glycine max*) (Additional file 1: Fig. S1). But something unusual observed when the vectors transformed into *Agrobacterium* strain EHA 105. The *Agrobacterium* transformed with pCB301-SC3 (#SC3) and pCB301-SC7 (#SC7) both showed the normal growth (though *Agrobacterium* transformed with #SC3 grows a little slower than that with #SC7), but that transformed with pCB301-SC15 (#SC15) failed to grow as observed 2 days after transformation (Fig. 1b). To further verify the phenomena and check if it is universal for different *Agrobacterium* strains, experiments were repeated with *Agrobacterium* strains GV3101, LBA4404 and K599 under the same condition. The growth of *Agrobacterium tumefaciens* strains (GV3101 and LBA4404) were similar with EHA105, that no visible colonies were observed after they were transformed with pCB301-SC15 (#SC15); while no *Agrobacterium rhizogenes* strain K599 colonies observed no matter which of the three plasmids transformed (Fig. 1b). These phenomena imply specific sequence presents in pCB301-SC15 (#SC15) inhibits the *Agrobacterium* growth much stronger than pCB301-SC3 (#SC3) and pCB301-SC7 (#SC7).

### Growth inhibiting sequence of *Agrobacterium* pCB301-SC15

**Fig. 2:**
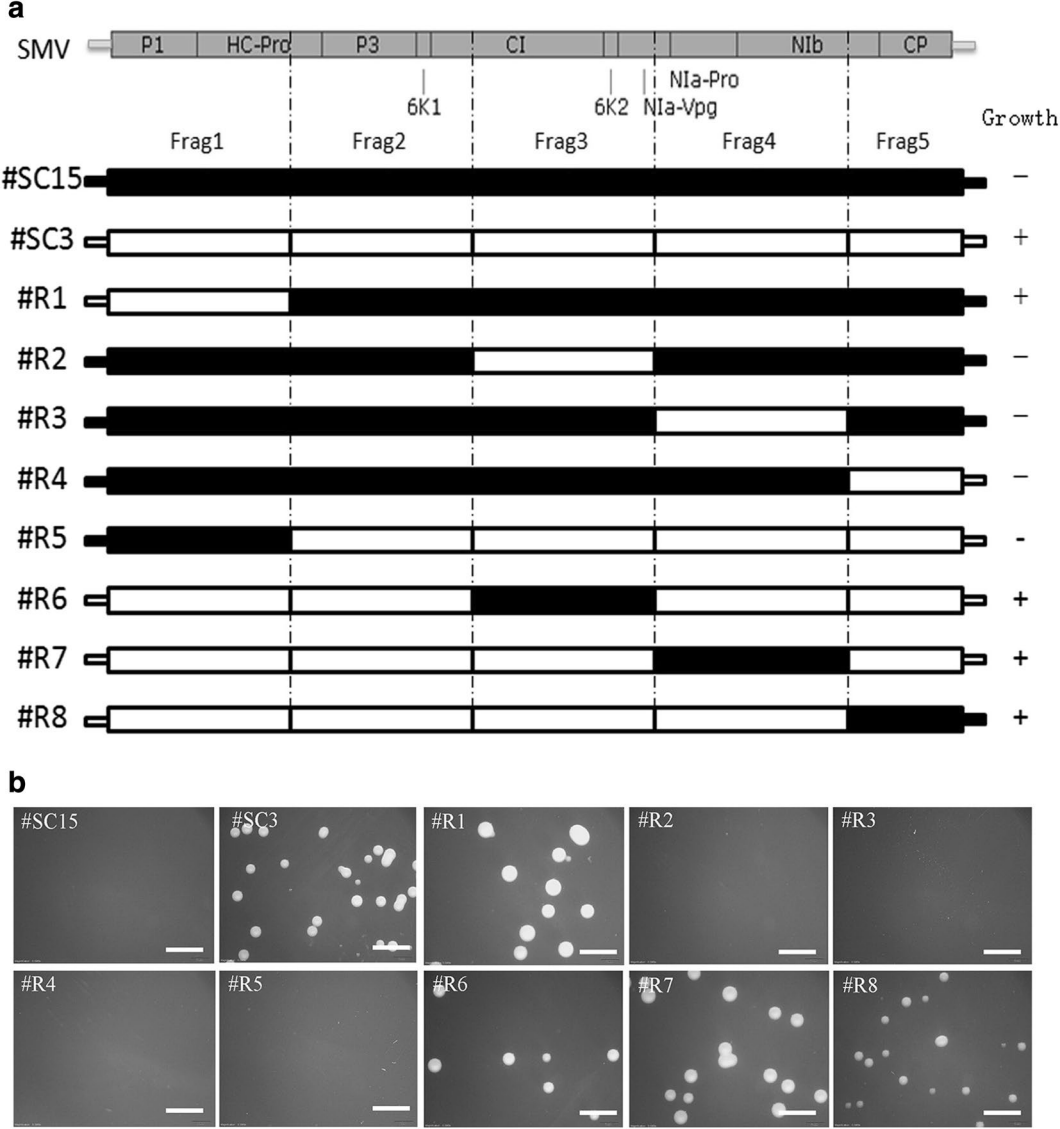
Growth of *Agrobacterium* after transformation with the recombinant clones of #SC15 and #SC3. **a** The recombinant position of #SC15 and #SC3 and the growth of *Agrobacterium* after the transformation. “+” indicates the colonies visible to the naked eye 2 days post-transformation, and “−” indicates those not. **b** Photographs of *Agrobacterium* growth after the transformation of each recombinant clone. Scale bars = 4 mm

To support and further illustrate findings of the present study, virus sequence of pCB301-SC15 and pCB301-SC3 were divided into five fragments (Frag 1 to 5). These fragments were amplified separately and used to construct a series recombinant vectors of the two previous clone vector (Fig. 2a). After the *Agrobacterium* EHA105 transformation, #R1, #R6, #R7, and #R8 clone vectors were found unable to inhibit the growth of *Agrobacterium*, while #R2, #R3, #R4 and #R5 cloning vectors still inhibited *Agrobacterium*’s growth (Fig. 2b). Fragments comparison analysis between different recombinant virus clone vectors, it become obvious that, the clone contained Frag1 from SC15 responsible for inhibited growth of *Agrobacterium* while the clone with Frag1 from SC3 approved the normal bacterium growth. In the SMV genome, Frag1 corresponds to 5′ UTR sequences, P1 protein-encoding sequences, and part of the HC-Pro protein encoding sequences.

**Fig. 3:**
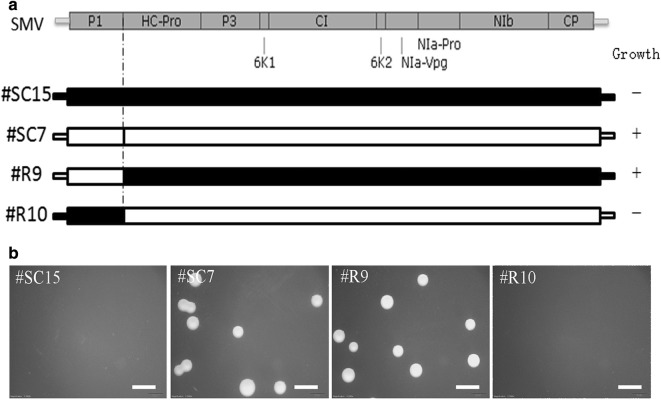
Growth of *Agrobacterium* after transformation with the recombinant clones of #SC15 and #SC7. **a** The recombinant position of #SC15 and #SC7 and the growth of *Agrobacterium* after transformation. “+” indicates the colonies visible to the naked eye 2 days post-transformation, “−” indicates those not. **b** Photographs of *Agrobacterium* growth after the transformation of each recombinant clone; scale bars = 4 mm

To further dig out the specific sequence and elaborate our findings, recombinant vectors that exchanged fragments between the SC15 and SC7 sequences (the recombination site was between P1 and HC-Pro; Fig. 3a) were constructed. Two days post-transformation, the clone with 5′ UTR and the P1-encoding sequences from SC7 and the remaining sequences from SC15 could no longer inhibit the growth of *Agrobacterium* strain EHA105. However, the clone with 5′ UTR and P1-encoding sequences from SC15 and the remaining sequences from SC7 showed inhibitory property (Fig. 3b). These findings demonstrated, whether or not SMV inhibits the growth of *Agrobacterium*, it depends on variation of fragment containing the 5′ UTR and the P1-encoding sequences.

**Fig. 4:**
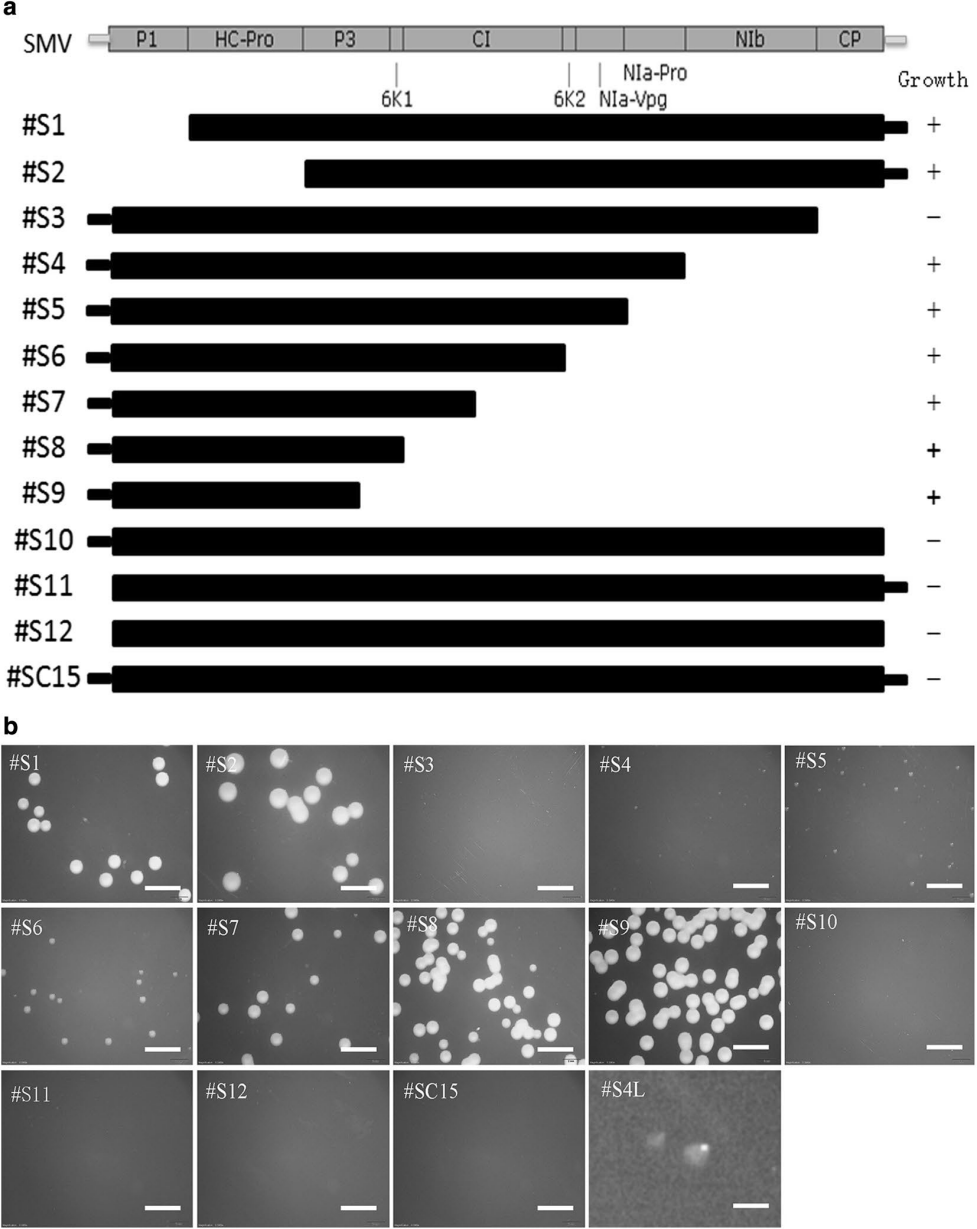
Growth of *Agrobacterium* after transformation with the truncated virus clone of #SC15. **a** The truncated position of #SC15 and the growth of *Agrobacterium* after the transformation. “+” indicates the colonies visible to the naked eye 2 days post-transformation, “−” indicates those not. **b** Photographs of *Agrobacterium* growth after the transformation of each truncated clone; photograph #S4L is a close-up of photograph #S4; the scale bar in #S4L is 0.4 mm but in the other photographs it is 4 mm

To elaborate the findings of present study and obtain the shortest sequence capable of inhibiting the growth of *Agrobacterium* strain EHA105, we constructed a series of clone vectors containing the truncated SC15 (Fig. 4a). We found that, #S1 and #S2 did not inhibit *Agrobacterium* growth so long as the P1 sequence was deleted (Fig. 4b), thus confirmed our findings. Furthermore, #S3, #S10, #S11, and #S12, which were clones without the UTR sequence or CP-encoding sequence, still prove the inhibition of *Agrobacterium* growth. So we can say, UTR and CP sequences not necessarily involved in *Agrobacterium*’s growth inhibition.

Finally, from the #S4 to #S9, we found that with a truncated 3′-end virus sequence, the growth of *Agrobacterium* became increasingly apparent, such that the growth inhibition effect was significantly reduced. As such, we gave the name SC15P to the smaller sequence (from P1 coding sequence to NIb coding sequence) that inhibits *Agrobacterium* growth.

### Application of the SC15 *Agrobacterium* growth inhibition sequence for efficient screening of recombinant clones

**Fig. 5:**
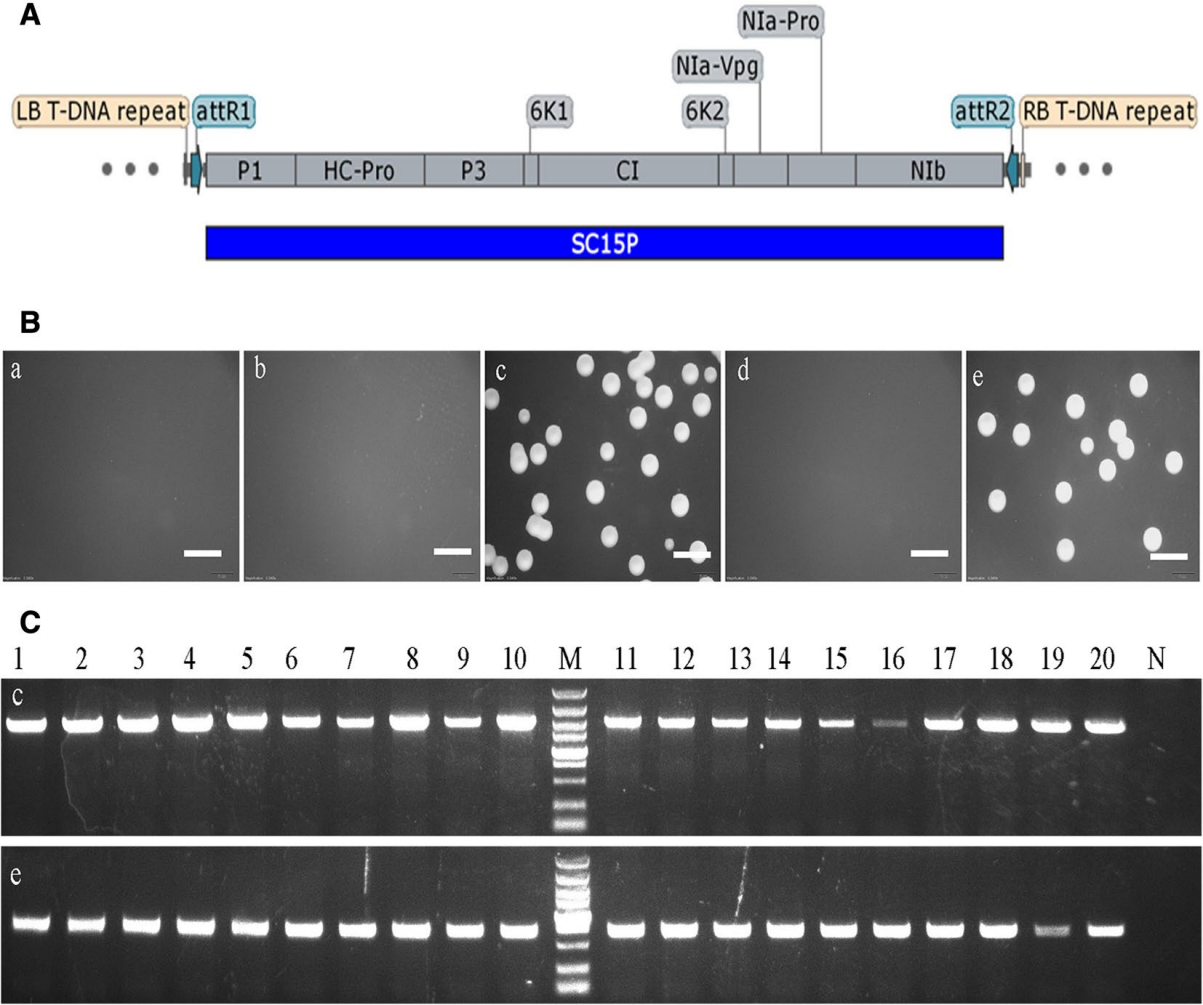
Application of the *Agrobacterium* growth inhibition sequence in screening recombinant clones. **A** Structure of the destination vector pCB301-*att*L-SC15P, which shows the elements between LB and RB; **B** Growth of *Agrobacterium* at 2 days post-transformation with pCB301-*att*L-SC15P (a), pDonor-Zeocin-CI (b), pDonor-Zeocin-Avh241 (c), LR recombination reaction product between pCB301-*att*L-SC15P and pDonor-Zeocin-CI (d), LR recombination reaction product between pCB301-*att*L-SC15P and pDonor-Zeocin- Avh241 (e); scale bars = 4 mm. **C** Identification of the colonies by PCR: 1 to 20 of (c) and (e) represent the colonies picked from the (c) and (e) plate in the B panel; N represents the negative control that used ddH_2_O as its template; the DNA marker is DL5000

To further support our findings, and check whether *Agrobacterium* growth inhibition sequence from SC15 applicable in screening recombinant clone directly in *Agrobacterium*, a destination vector pCB301-*att*L-SC15P based on the LR recombinant reaction was constructed (Fig. 5a). We replaced the sequences between LB and RB in the vector pCB301 by the *att*L1 and *att*L2 sequences, then inserted the fragment SC15P between the *att*L1 and *att*L2 sequences. Two days post-transformation, the results further supports our findings, showed that pCB301-*att*L-SC15P inhibit the growth of *Agrobacterium* (Fig. 5b). Later on, pCB301-*att*L-SC15P used in LR recombination reaction with pDonor-CI and pDonor-Avh241, products were transformed into *Agrobacterium* directly (Additional file 1: Fig. S2). Twenty *Agrobacterium* colonies were selected from each plate and confirmed the presence of inserted sequence by PCR and by sequencing. All chosen single colonies contained the correct recombination clones (Fig. 5b).

## Discussion

The only *Agrobacterium* growth inhibition gene SacB which has been reported to be used in Agrobacterium screening as a negative factor is derived from *Bacillus subtilis* (Chambert and Petit-Glatron 1989; Quandt and Hynes 1993; Traore and Zhao 2011). The levansucrase encoded by SacB is involved in the hydrolysis of sucrose and the synthesis of levan. But the levan is toxic to most Gram-negative bacterium including *Agrobacterium* as it can’t be metabolized by them. So the growth of *Agrobacterium* can be suppressed on the medium containing high concentration of sucrose. Here, it is the first time that we found the plant virus fragment can also inhibit the growth of *Agrobacterium*. We remain unsure at which step the real physiological inhibition of *Agrobacterium* actually happens. Put differently, we do not know whether it is the protein encoded by the sequence or the transcripted RNA, or the DNA itself limit *Agrobacterium* growth. If the functional component is not the DNA sequence per se, then how does the viral DNA sequence start transcription and translation without a known promoter? But it is not difficult to speculate that the reason underlying the viral DNA sequence is exactly different from that of SacB. Although this experiment has proved that the polymorphisms of P1 coding sequence (Figs. 2, 3, Additional file 1: Fig. S3) of SMV strains lead different effects on *Agrobacterium*, the molecular mechanism behind it is still untouched. Furthermore, we only know that the lack of other protein encoding sequences reduces the ability of SC15 to suppress *Agrobacterium* growth (Fig. 4); but what role the other sequences play is unknown. For all the reasons above, we encourage interested peers to continue explore the secrets of it.

As we all know, in the process of vector construction and screening, false positives usually occur because of vector self-ligation, or other factors associated with traditional restriction-ligation method. But, by applying a negative selection factor, the experiment is more easily and efficiently executed (Bernard et al. 1994; Wu et al. 2008). The most famous example of this is the Gateway cloning system, which uses the *ccd*B toxic gene as the negative selection factor (Curtis and Grossniklaus 2003). In our experiment reported here, by taking the Gateway cloning system as a basement, we substituted the *ccd*B gene with an SMV fragment as the new negative factor to construct a new destination vector (Fig. 5, Additional file 1: Fig. S2). And the efficient and accurate recombination reactions between the new destination vector and donor vector show the feasibility of using this SMV fragment as a negative selection factor.

In the study of plant gene function, *Agrobacterium* are widely used in the experiments of protein subcellular localization, protein interaction, gene knockout and genetic complementation. In order to carry out high throughput experiments, many Gateway-compatible plant binary vector sets were constructed by different laboratories and also widely used (Curtis and Grossniklaus 2003; Earley et al. 2006; Nakagawa et al. 2007). With these vector sets, researchers could insert interest genes into different plasmid easily, but this procedures still need to be done in *E. coli* before transformed into *Agrobacterium*. As the successful attempt on directly screening recombinant clones in *Agrobacterium*, we can also modify the available plant binary vectors to make the experiments for plant gene function studies simpler and less time-consuming. If the plasmid to be modify already compatible with LR reaction, just replace the fragment between *att*R1 and *att*R2 with SC15P; if it is the common plasmid with multiple cloning sites, *att*R1 and *att*R2 site sequence also need to be insert, and sequence put in should be examined carefully to avoid frameshift and pre-termination of original open reading frame. This kind of modification can be done with a few skills by any molecular biology laboratory.

Based on the results obtained in this study, we showed that the SC15P (fragment from SMV strain SC15) inhibited the growth of *Agrobacterium* greatly. As an example, SC15P was used as negative selection marker successfully in *Agrobacterium* which transformed with LR reaction products directly, made the procedure more time-efficient and simpler. In the future, more effort should be put into understanding the underlying molecular mechanism of *Agrobacterium* inhibition, which will be helpful to shorten the fragment and promote its usage in vector construction.

## Additional file

**Additional file 1: Table S1.** Primers used for vectors construction. **Figure S1.** Phenotypes of Soybean cultivar Nannong 1138-2 leaves infected by different virus clones. **Figure S2.** Apply SC15P in efficient recombinant chones screening in *Agrobacterium.* **Figure S3.** P1 coding sequences alignment of three SMV strains.

## Data Availability

All data generated or analyzed during this study are included in the figures and tables. Any material used in this study is available for research purposes upon request.
