# Supplementary material for: Discovery of the Agrobacterium growth inhibition sequence in virus and its application to recombinant clone screening
Source: AMB Express. 2019 Jul 24;9:116. doi: 10.1186/s13568-019-0840-3 (PMC6656845; doi:10.1186/s13568-019-0840-3)
Supplement: Supplementary file 1 — Additional file 1: Table S1. Primers used for vectors construction. Figure S1. Phenotypes of Soybean cultivar Nannong 1138-2 leaves infected by different virus clones. Figure S2. Apply SC15P in efficient recombinant chones screening in Agrobacterium. Figure S3. P1 coding sequences alignment of three SMV strains. [file 13568_2019_840_MOESM1_ESM.pdf]

Supplementary Material to:

Discovery of the *Agrobacterium* Growth Inhibition Sequence in Virus and its Application to Recombinant Clone Screening

Jinlong Yin[1], Hui Liu[1], Wenyang Xiang[1], Tongtong Jin[1], Dongquan Guo[2], Liqun Wang[1 §], Haijian Zhi[1 §]

[1]National Center for Soybean Improvement, National Key Laboratory for Crop Genetics and Germplasm Enhancement, Nanjing Agricultural University, Weigang 1, Nanjing 210095, P.R. China

[2]Jilin Academy of Agricultural Sciences, Changchun 130033, P.R. China

[§]Corresponding author 1: Haijian Zhi

[§]Corresponding author 2: Liqun Wang

Authors' e-mail address list

Jinlong Yin：yinjinlong0000@126.com

Hui Liu：18305189228@163.com

Wenyang Xiang:897503352@qq.com

Tongtong Jin: shona126@sina.com

DongQuan Guo：xzgdq@126.com

Liqun Wang： wanglq1124@126.com

Haijian Zhi：zhj@njau.edu.cn

Table S1. Primers used for vectors construction.

Vector	Fragment	Size (bp)	Primer name	Primer sequence
SC3	1a	1352	SMV-frag1a-F	tgacgcacaatcccactatccttcgcaagacccttcctctatataaggaagttcatttcatttggagaggAAATTAAAACTACTCATAAAGACAACAAAC
			SMV-frag1a-R	GGAATTGTTTATACTCCTCCCAACT
	2a	2494	SMV-frag2a-F	CATGTGGAGAATCATGAATGCACCA
			SMV-frag2a-R	CATCCTCACCCATTGTGCTG
	3a	2229	SMV-frag3a-F	CAGCAACTCAATTGCAACTTGAGAAAATTG
			SMV-frag3a-R	GTATCTGCCTCTTCTTTCCTTGAGTTGATAC
	4a	3370	SMV-frag4a-F	GTTGACATCAACAACCTGCACAATGTTG
			SMV-frag4a-R	GCTCATATTCATCTTTAACTGCATTGTACCAC
	5a[a]	1156	SMV-frag5a-F	GGTGGTTCCGCGTTTGCAGAAGATTAC
			SMV-frag5a-R-1	GGAGGTGGAGATGCCATGCCGACCCTTTTTTTTTTTTTTTTTTTTTTTTTTTTTTTTTTTTTTTTTTTTTTTTTTTTTTTAGGACAACAAACATTGCCG
			SMV-frag5a-R-2	gctctccctgacccAGTGGCTCTCCCTTAGCCATCCGAGTGGACGTGCGTCCTCCTTCGGATGCCCAGGTCGGACCGCGAGGAGGTGGAGATGCCATGC
			SMV-frag5a-R-3	accggcaacaggattcaatcttaagaaactttattgccaaatgtttgaacgatcggggaaattcgagctctccctgacccAG
SC7	1b	1812	SMV-frag1c-F-1	tgacgcacaatcccactatccttcgcaagacccttcctctatataaggaagttcatttcatttggagaggAAATTAAAACAACTCATAAAGACAAC
			SMV-frag1a-2R	GATGTCTCAATCACTTTCTTCACATAC
	2b	2500	SMV-frag2a-F	CATGTGGAGAATCATGAATGCACCA
			SMV-frag2a-2R	CTTTAACATCCTCACCCATTGTG
	3b	2229	SMV-frag3a-F	CAGCAACTCAATTGCAACTTGAGAAAATTG
			SMV-frag3a-R	GTATCTGCCTCTTCTTTCCTTGAGTTGATAC
	4b	3370	SMV-frag4a-F	GTTGACATCAACAACCTGCACAATGTTG
			SMV-frag4a-R	GCTCATATTCATCTTTAACTGCATTGTACCAC
	5b[a]	1162	SMV-frag5a-F	GGTGGTTCCGCGTTTGCAGAAGATTAC

			SMV-frag5a-R-2	gctctccctgacccAGTGGCTCTCCCTTAGCCATCCGAGTGGACGTGCGTCCTCCTTCGGATGCCCAGGTCGGACCGCGAGGAGGTGGAGATGCCATGC
			SMV-frag5a-R-3	accggcaacaggattcaatcttaagaaactttattgccaaatgtttgaacgatcggggaaattcgagctctccctgacccAG
			SMV-frag5a-R-3	accggcaacaggattcaatcttaagaaactttattgccaaatgtttgaacgatcggggaaattcgagctctccctgacccAG
SC15	1c	1351	SMV-frag1a-F	tgacgcacaatcccactatccttcgcaagacccttcctctatataaggaagttcatttcatttggagaggAAATTAAAACTACTCATAAAGACAACAAAC
			SMV-frag1-R(6067)	GGAATTGTTTATACTCTTCCCAACT
	2c	2500	SMV-frag2a-F	CATGTGGAGAATCATGAATGCACCA
			SMV-frag2a-2R	CTTTAACATCCTCACCCATTGTG
	3c	2229	SMV-frag3-F(6067)	CAGTAACTCAATTACAACTTGAGAAAATCG
			SMV-frag3-R(6067)	GTATTTGCCTCTTCTTTCCTTGAGTTGATAC
	4c	3370	SMV-frag4a-F	GTTGACATCAACAACCTGCACAATGTTG
			SMV-frag4-R(6067)	GTTCATATTCATCTTTGACTGCATTGTACCAT
	5c[a]	1162	SMV-frag5-F(6067)	GGTGGTTCCACGCCTGCAGAAGATTAC
			SMV-frag5a-R-1	GGAGGTGGAGATGCCATGCCGACCCTTTTTTTTTTTTTTTTTTTTTTTTTTTTTTTTTTTTTTTTTTTTTTTTTTTTTTTTTTTAGGACAACAAACATTGCCG
			SMV-frag5a-R-2	gctctccctgacccAGTGGCTCTCCCTTAGCCATCCGAGTGGACGTGCGTCCTCCTTCGGATGCCCAGGTCGGACCGCGAGGAGGTGGAGATGCCATGC
			SMV-frag5a-R-3	accggcaacaggattcaatcttaagaaactttattgccaaatgtttgaacgatcggggaaattcgagctctccctgacccAG
#R1<br>#R2<br>#R3<br>#R4<br>#R5<br>#R6<br>#R7<br>#R8	1d	2019	pCB301-REC-F	GGAAGTTCATTTCATTTGGAGAGG
			153-1R	GGCGCTTTGTAGGACTCTTG
	2d	2113	153-1F	CAAGAGTCCTACAAAGCGCC
			153-2R	CGCAAACCCACTTGTCATGAC
	3d	2038	153-2F	GTCATGACAAGTGGGTTTGCG
			153-3R	CAACCTTGAGTGCTTCCTCTG
	4d	2198	153-3F	CAGAGGAAGCACTCAAGGTTG
			153-4R	CTCAGTGCGATGCATAAGCTC
	5d	1494	153-4F	GAGCTTATGCATCGCACTGAG

			pCB301-REC-R	ATTCGAGCTCTCCCTGACCC
#R9	1e	1558	pCB301-REC-F	GGAAGTTCATTTCATTTGGAGAGG
			SMV7-15R	AAACTGAGCTTCAGGATTTTGGGAATAATGCTGGATGTCCCTCATGCTC
	2e	8765	SMV7-15F	GAGCATGAGGGACATCCAGCATTATTCCCAAAATCCTGAAGCTCAGTTT
			pCB301-REC-R	ATTCGAGCTCTCCCTGACCC
#R10	1f	1152	pCB301-REC-F	GGAAGTTCATTTCATTTGGAGAGG
			SMV15-7R	AAACTGAACCTCAGGATTTTGGGAGTAGTGCTGAATATCATCCATGTTT
	2f	8765	SMV15-7F	AAACATGGATGATATTCAGCACTACTCCCAAAATCCTGAGGTTCAGTTT
			pCB301-REC-R	ATTCGAGCTCTCCCTGACCC
#S1	1g	8697	HCPRO-recF	GGAAGTTCATTTCATTTGGAGAGGATGTCCCAAAATCCTGAAGCTCAG
			pCB301-REC-R	ATTCGAGCTCTCCCTGACCC
#S2	1h	7326	P3-REC-F	GGAAGTTCATTTCATTTGGAGAGGATGGGTGAAGTGCAACAGAGAATG
			pCB301-REC-R	ATTCGAGCTCTCCCTGACCC
#S3	1i	8561	NIb-REC-R	ATTCGAGCTCTCCCTGACCCTTGTAAGGACACTGATTCACAAC
			pCB301-REC-F	GGAAGTTCATTTCATTTGGAGAGG
#S4	1j	7013	NIaPro-REC-R	GGAAGTTCATTTCATTTGGAGAGGATGCCCTTGAACTGTCACTGTGTTTCC
			pCB301-REC-F	GGAAGTTCATTTCATTTGGAGAGG
#S5	1k	6281	NIaVpgR-REC-R	GGAAGTTCATTTCATTTGGAGAGGTTCCATTTCAACTCTTTCTTTGG
			pCB301-REC-F	GGAAGTTCATTTCATTTGGAGAGG
#S6	1l	5713	6K2-REC-R	ATTCGAGCTCTCCCTGACCCTTGAGTTGATACTGGTTCACGT
			pCB301-REC-F	GGAAGTTCATTTCATTTGGAGAGG
#S7	1m	4112	pCB301-REC-F	GGAAGTTCATTTCATTTGGAGAGG
			SMV-poi2-R	GAAATTCGAGCTCTCCCTGACCCCGCAAACCCACTTGTCATGAC
#S8	1n	3650	6K1-REC-R	ATTCGAGCTCTCCCTGACCCCTGCACTTTAACATCCTCACCC
			pCB301-REC-F	GGAAGTTCATTTCATTTGGAGAGG
#S9	1o	2958	pCB301-REC-F	GGAAGTTCATTTCATTTGGAGAGG
			SMV-poi1-R	GAAATTCGAGCTCTCCCTGACCCGCTTAATGCGCGCCATTCCTG
#S10	1p	9356	pCB301-REC-F	GGAAGTTCATTTCATTTGGAGAGG

			SMV-CP-REC-R	ATTCGAGCTCTCCCTGACCCCTGCTGTGGGCCCATGCCC
#S11	1q	9624	pCB301-REC-R	ATTCGAGCTCTCCCTGACCC
			SMV-P1-REC-F	ATTCGAGCTCTCCCTGACCCATGGCAACAATCATGATTGGAAG
#S12	1r	9201	SMV-P1-REC-F	ATTCGAGCTCTCCCTGACCCATGGCAACAATCATGATTGGAAG
			SMV-CP-REC-R	ATTCGAGCTCTCCCTGACCCCTGCTGTGGGCCCATGCCC
pCB301-attL-ccdB	1s	1796	attL1-301REC	GACGCTTAGACAACTTAATAACACGGGGGACTCTAATCAAACAAG
			attL2-301REC	CACTGATAGTTTAAACTGAAGGCGGATCCTCTAGATCGAACCAC
	2s	5461	301-frag-F	CGCCTTCAGTTTAAACTATCAGTG
			301-frag-R	GTGTTATTAAGTTGTCTAAGCGTC
pCB301-attL-SC15P	1t	5811	attR1-F	GTAAAGCCTGGGGTGCCTA
			attR2-R	CTTATACACAGCCAGTCTGCAGGTC
	2t	8445	NIb-attr2-recR	GCAGACTGGCTGTGTATAAGTTGTAAGGACACTGATTCACAACAATC
			P1-attr1-recF	TAGGCACCCCAGGCTTTACATGGCAACAATCATGATTGGAAGC

Note: [a] indicates that we did the second and third PCR to elongation the PCR product used the former PCR product as template.

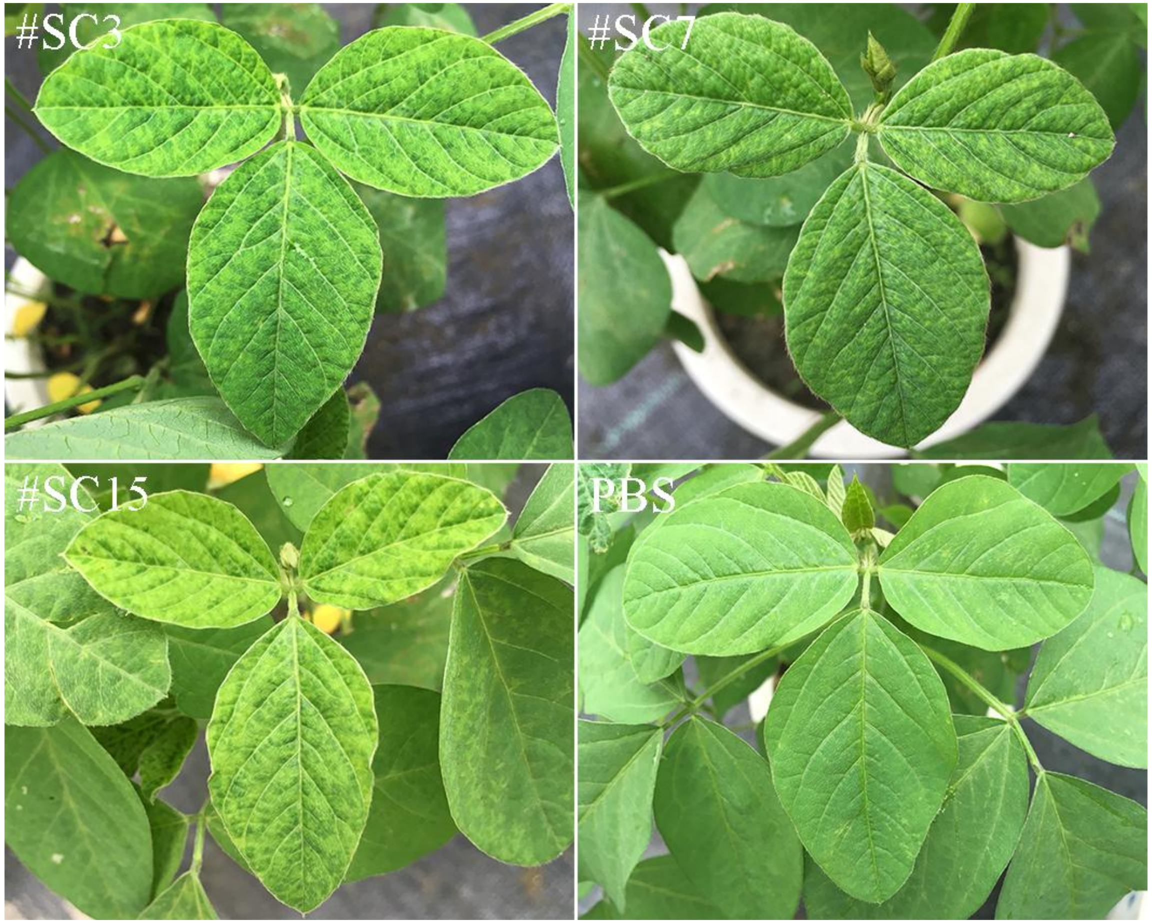

Fig. S1. Phenotypes of Soybean cultivar Nannong 1138-2 leaves infected by different virus clones.
Photographs were taken 2 weeks after inoculated #SC3, #SC7, #SC15 plasmid or PBS (phosphate buffer solution, as negative control)

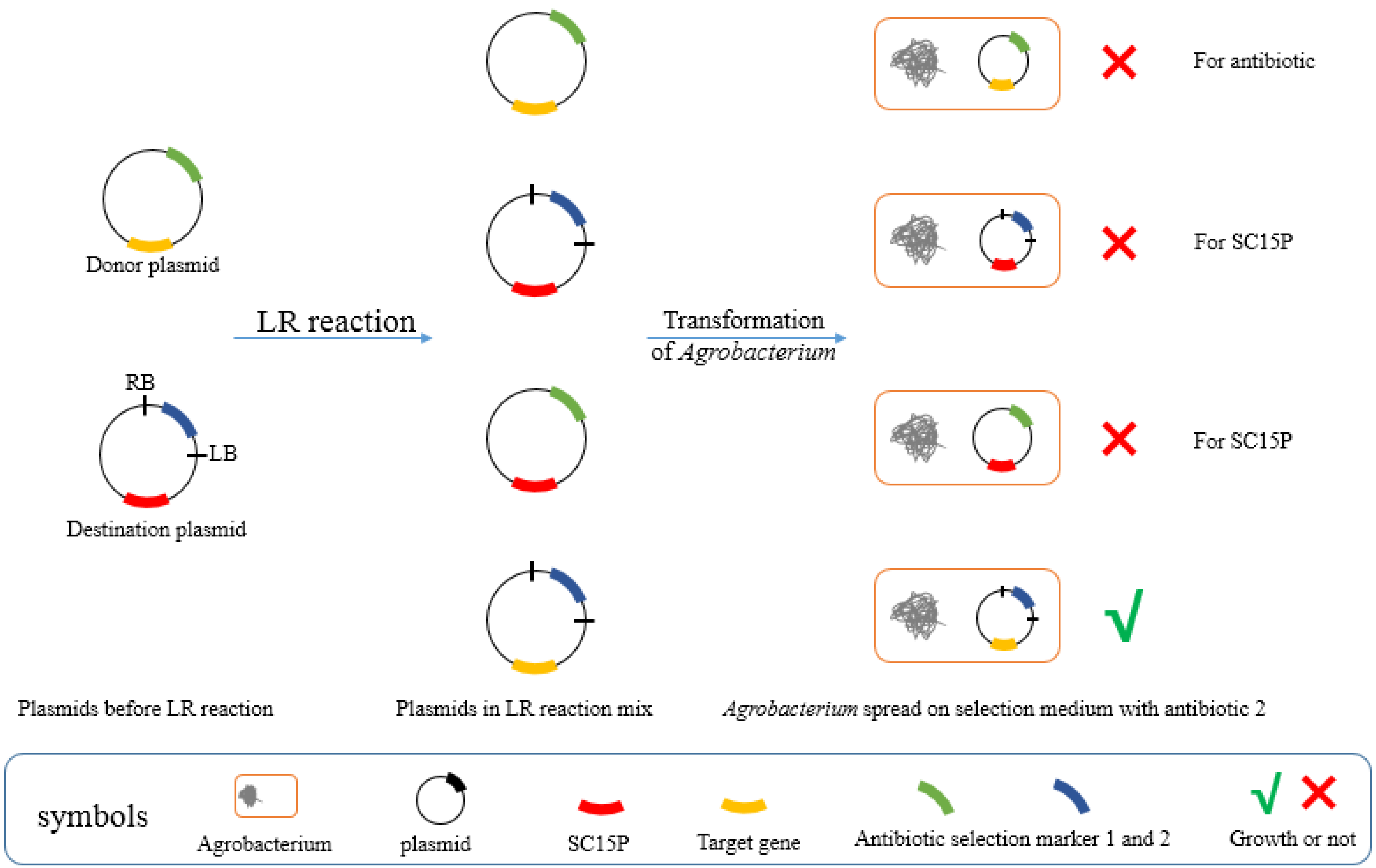

Fig. S2. Apply SC15P in efficient recombinant chones screening in *Agrobacterium*.

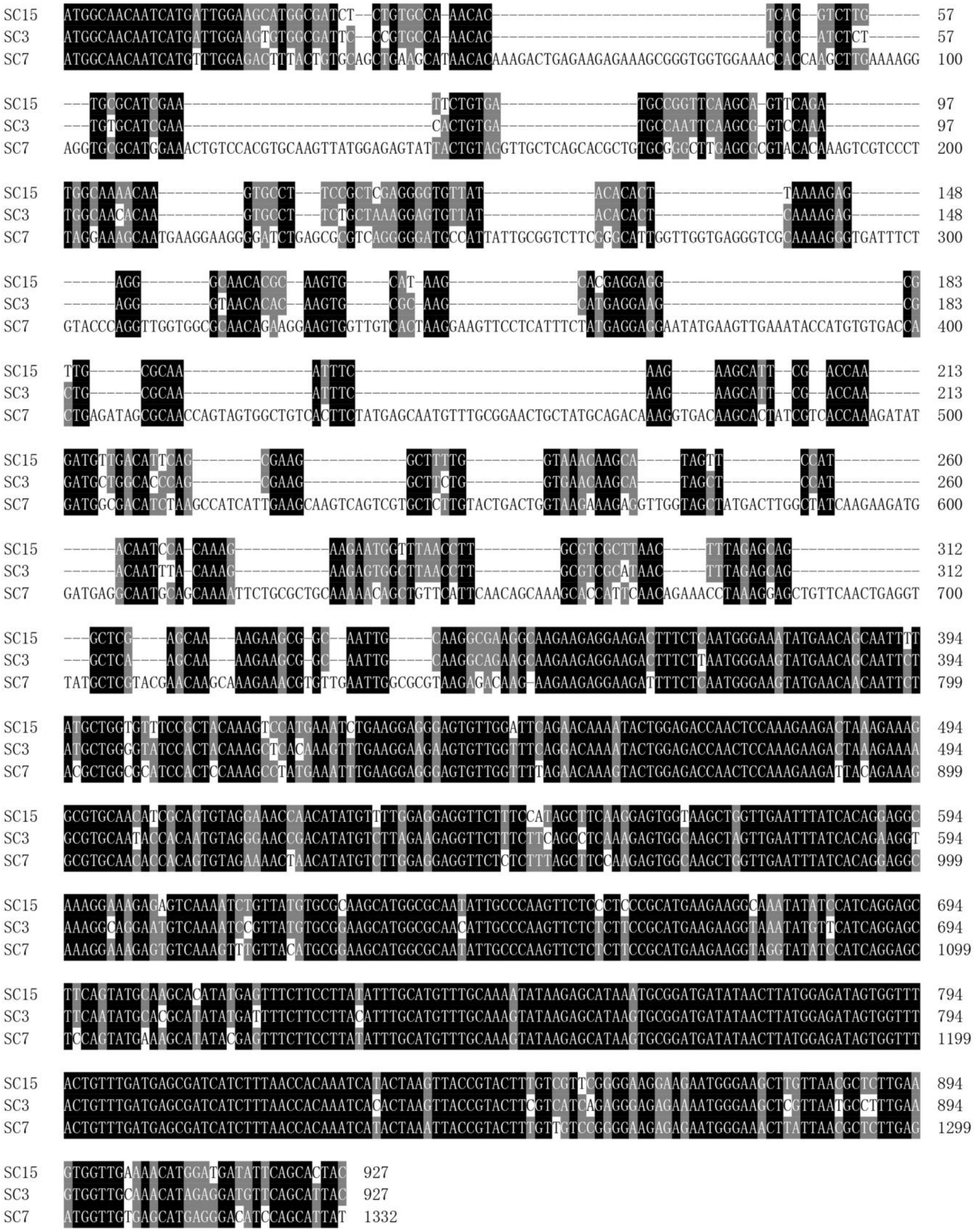

Fig. S3. P1 coding sequences alignment of three SMV strains.

Nucletide aignments were performed using Clustal omega (http://www.ebi.ac.uk/Tools/msa/clustalo)
